# Pregnancy After Cardiac Surgery

**DOI:** 10.7759/cureus.31133

**Published:** 2022-11-05

**Authors:** Nidhi Makhija, Surekha Tayade, Hard Tilva, Arzoo Chadha, Utkarsh Thatere

**Affiliations:** 1 Department of Obstetrics and Gynaecology, Jawaharlal Nehru Medical College, Datta Meghe Institute of Medical Sciences, Wardha, IND; 2 Department of Medicine, Jawaharlal Nehru Medical College, Datta Meghe Institute of Medical Sciences, Wardha, IND

**Keywords:** anticoagulants, warfarin, warfarin in pregnancy, anticoagulants in pregnancy, valve replacements, cardiac disease, pregnancy

## Abstract

Women with native heart valve disease who are considering getting pregnant should have a complete risk estimation to determine whether an intervention is required prior to becoming pregnant and, if so, to determine when it should be performed and what kind of surgical therapy will be used. Pregnancy is linked to early and late structural valve degeneration in women who have bioprostheses, suggesting a high reoperation rate. A mechanical valve during pregnancy increases the risk of maternal complications such as valve thrombosis and mortality. The claim that women with defective hearts should not become pregnant was driven by the high maternal death rate among cardiac patients who became pregnant. A preoperative anticoagulation therapy trial helped women scheduled for valve replacement to acquire complete information as to the choice of the prosthetic device. Integrated risk stratification scheme for pregnant patients with valvular heart disease, with WHO classification and an algorithmic approach to both preconception counseling and anticoagulation strategy as outlined here, as well as early referral to a cardiologist with expertise in the management of cardiac disease and pregnancy for these complex patients is recommended. However, in reality, some women present while pregnant and valve disease needs to be managed, balancing maternal outcome and fetal risk. In general, optimizing the hemodynamic situation of the mother is also beneficial to the fetus. However, cardiac surgery carries a high risk for the fetus. No anticoagulant regimen can be said to be entirely safe for use during pregnancy, as there is a degree of risk with each regimen. Therefore, this review has been done to find appropriate management for women dealing with such conditions.

## Introduction and background

Heart disease affects pregnant women between 1% and 4% of the time. Heart disease is the main factor in indirect obstetric fatalities, complicating more than 1% of pregnancies and contributing to 15% of maternal mortality [1]. The rate of maternal death has not even decreased in wealthy nations. Undiagnosed heart disease is aggravated by pregnancy, which also causes pregnant women to manifest overt symptoms. The hemodynamic decompensation stage is frequently reached before cardiac disease in pregnant women is discovered due to a lack of medical knowledge and awareness. The need for heart surgery is now unavoidable [1]. The general rule is that heart surgery should be postponed until after birth, ideally for six weeks. However, early pregnancy cardiac surgical intervention is required when these pregnant women experience overt symptoms. A critical first step in management is the formation of a multidisciplinary team (MDT).

Unfractionated heparin (UFH), low molecular weight heparin (LMWH), and oral anticoagulants (coumarin derivatives) are available as treatments. If used throughout pregnancy, coumarin derivatives lower the risk of thromboembolic complications in pregnant women to 3.7%, with a 0.5-1.8% chance of maternal mortality. With a reported 12% incidence of late fetal death and a 6.4% prevalence of congenital abnormalities, its usage must be restrained due to its likely harmful effects on the fetus. As a result, many experts now recommend switching to heparin instead of coumarin derivatives, at least during the first trimester. The high risk of treatment failure with reports of maternal death from thrombosed valves is one disadvantage of UFH.

The main maternal result was outlined as a combination of systemic thromboembolism, prosthetic valve failure, and maternal death. Abnormal valve performance that results in a clinically significant consequence, such as heart failure, an arrhythmia, or reoperation, was described as prosthetic valve failure. Any systemic arterial thrombotic event, such as a stroke or transient ischemic attack, was referred to as thromboembolism. A combination of spontaneous abortion, fetal death, and the existence of any congenital abnormality were known as the primary fetal fate.

Radiation exposure during diagnostic or therapeutic operations on pregnant patients may result in elevated levels of anxiety in both the patients and the medical staff. This can occasionally lead to pregnancy termination. Ionizing radiation can cause cell death, teratogenic effects, carcinogenesis, and genetic damage, among other negative consequences. However, given the levels required for the majority of diagnostic and therapeutic procedures, these effects are not seen. Since the fetus is shielded from direct radiation during cardiac operations, the risk to the unborn child is significantly reduced. Radiation exposure should be avoided wherever feasible, notwithstanding the negligible dangers. On the other hand, the mother can be comforted if an operation is truly essential and there are no other options. Although abdominal shielding only reduces the exposure to the fetus by 2%, it is advised. The abdomen region should not be exposed to direct radiation in any way. The first trimester, when organogenesis has finished but the uterus is still small, is the optimal time to do invasive treatments.

Here, we review the literature currently available on this concerning issue and its management.

## Review

Methods

**Figure 1:**
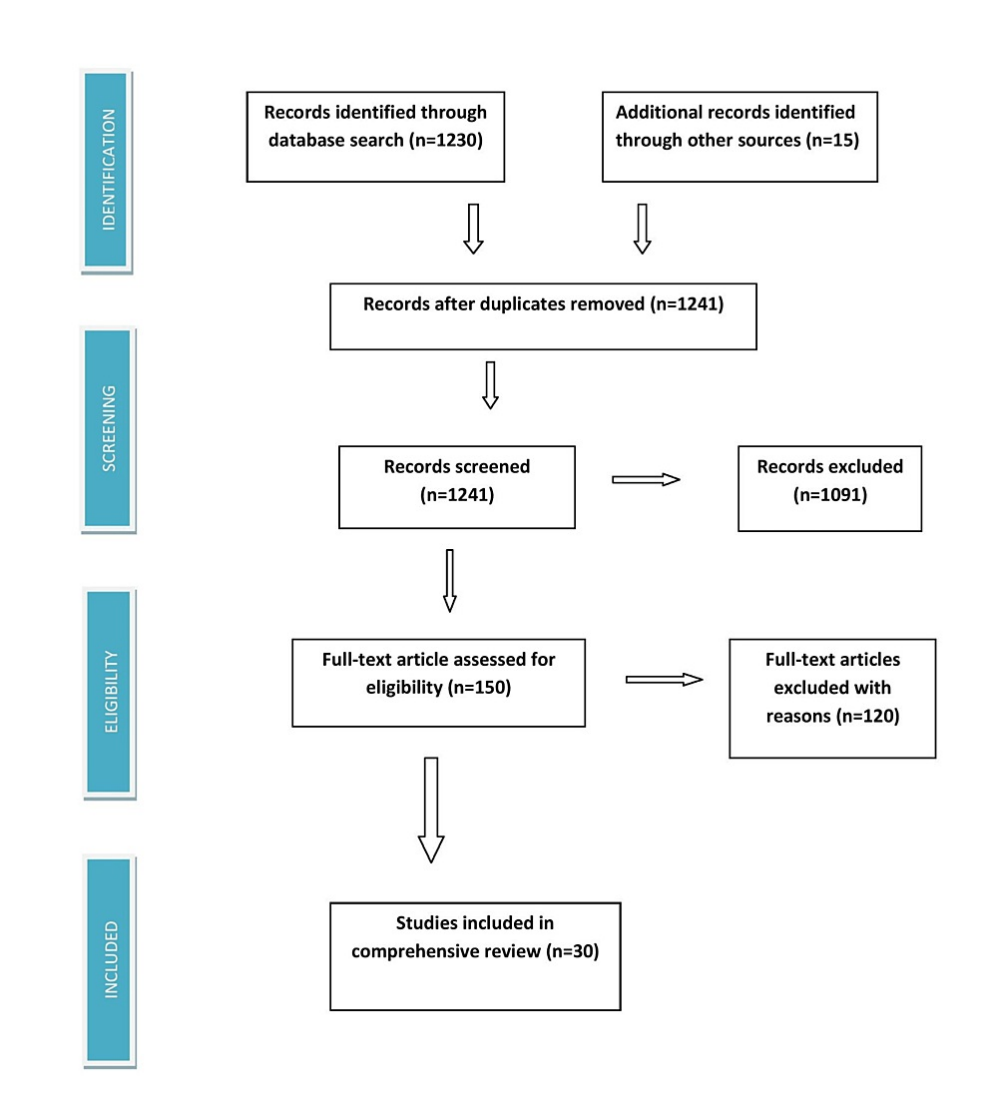
PRISMA flow diagram PRISMA = Preferred Reporting Items for Systematic Reviews and Meta-Analyses.

Using Medical Subject Heading (MeSH) keywords, including pregnancy, cardiac surgery, rheumatic heart disease, and prosthetic valves, we searched PubMed, MEDLINE, Embase, ISI Web of Science, and Google Scholar, and we discovered 1,241 articles between 1970 and 2022. When we limited our search to the years 2000 to 2022, we were able to obtain 30 pertinent publications. Below is a diagram of how data collecting works (Figure 1). A tabulated depiction of all relevant articles is given below in Table 1.

**Table 1:** Table depicting the relevant articles in this study

Sr. No.	Year of publication	Authors	Title	Sample size
1.	2014	Youhao You et al. [2]	Cardiac surgery under cardiopulmonary bypass in pregnancy: report of four cases	4
2.	2019	Jamie LR Romeo et al. [3]	Influence of pregnancy on long-term durability of allografts in right ventricular outflow tract	196
3.	2011	Luca S De Santo et al. [4]	Mechanical aortic valve replacement in young women planning on pregnancy	40
4.	2003	Renato T Arnoni et al. [5]	Risk factors associated with cardiac surgery during pregnancy	74
5.	2014	Snehalata Basude et al. [6]	Pregnancy outcome and follow-up cardiac outcome in women with aortic valve replacement	32
6.	2003	Walkiria Samuel Avila et al. [7]	Pregnancy in patients with heart disease: experience with 1,000 cases	1000
7.	2002	Adil AM Al-Lawati et al. [8]	Pregnancy and mechanical heart valves replacement; dilemma of anticoagulation	63
8.	2005	Uri Elkayam et al. [9]	Valvular heart disease and pregnancy	51
9.	2013	Adeline Fuchs et al. [10]	Valve-in-valve and valve-in-ring transcatheter mitral valve implantation in Young women contemplating pregnancy	12
10.	2010	Martin Sillesen et al. [11]	Pregnancy with prosthetic heart valves — 30 years’ nationwide experience in Denmark	356
11.	2022	Michael Nanna et al. [12]	Pregnancy complicated by valvular heart disease: an update	-
12.	2012	B Mazibuko et al. [13]	An audit of pregnant women with prosthetic heart valves at a tertiary hospital in South Africa: a five-year experience	1021
13.	2014	Karen Sliwa et al. [14]	Management of valvular disease in pregnancy: a global perspective	-
14.	2011	Vanita Suri et al. [15]	Mechanical valve prosthesis and anticoagulation regimens in pregnancy: a tertiary center experience	40
15.	2017	Zachary L Steinberg et al. [16]	Maternal and fetal outcomes of anticoagulation in pregnant women with mechanical heart valves	800
16.	2000	Carlos Ibarra-Perez et al. [17]	The course of pregnancy in patients with artificial heart valves	25
17.	2021	Susy Kotit et al. [1]	Cardiovascular adverse events in pregnancy: a global perspective	-
18.	2022	Molly M Daughety et al. [18]	Management of anticoagulation in pregnant women with mechanical heart valves	-
19.	2015	Yoshio Misawa et al. [19]	Valve‑related complications after mechanical heart valve implantation	1914
20.	2003	SZ Bhutta et al. [20]	Pregnancy following cardiac surgery	170
21.	2008	Walkiria Samuel Ávila et al. [21]	Maternal-fetal outcome and prognosis of cardiac surgery during pregnancy	40
22.	2012	PG Pieper et al. [22]	Cardiac surgery and percutaneous intervention in pregnant women with heart disease	-
23.	2017	Karolina Adam et al. [23]	Pregnancy in women with cardiovascular diseases	-
24.	2022	Gijs J van Steenbergen et al. [24]	Timing of cardiac surgery during pregnancy: a patient-level meta-analysis	-
25.	2014	Shi-Min Yuan et al. [25]	Infective endocarditis during pregnancy	30
26.	2022	H Singh et al. [26]	Pregnancy after surgical correction of tetralogy of Fallot	40
27.	2013	SM Yuan et al. [27]	Indications for cardiopulmonary bypass during pregnancy and impact on fetal outcomes	150
28.	2013	Titia PE Ruys et al. [28]	Pregnancy and delivery in cardiac disease	-
29.	2013	Martin T Yates et al. [29]	Perioperative management and outcomes of aortic surgery during pregnancy	11
30.	2004	SA Thorne et al. [30]	Pregnancy in heart disease	-

According to the WHO, “cardiovascular diseases (CVDs) are the leading cause of death globally, accounting for 17.9 million deaths annually.” Heart and blood vessel conditions known as CVDs include conditions like coronary heart disease, cerebrovascular disease, rheumatic heart disease, and other ailments [31].

Premature deaths can be avoided by identifying people who are most susceptible to CVDs and making sure they receive proper care [31]. To guarantee that people in need receive treatment and counseling, access to noncommunicable disease medications and core health technology in all primary healthcare institutions is crucial.

Valves and their diseases

The four heart valves are the mitral valve, tricuspid valve, pulmonary valve, and aortic valve. Each valve has flaps; for the aortic valve and pulmonary valve, they are called cusps, and for the mitral valve and tricuspid valve, they are referred to as leaflets. Each heartbeat should cause these flaps to open and shut once. Blood flow via the heart to the body is disrupted when valves do not open or seal appropriately.

Mitral Stenosis

Pregnancy-induced tachycardia, increased stroke volume, and impaired diastolic flow via the stenotic valve combine to increase left atrial pressure and dyspnea, and may even lead to pulmonary edema. Atrial fibrillation compromises the patient much further. In addition, right ventricular failure could occur as a result of secondary pulmonary hypertension [30].

Bicuspid Aortic Valve Stenosis

Aortic stenosis in young people is typically asymptomatic. Angina, pulmonary edema, new ECG alterations, disproportionate dyspnea or tachycardia, a decrease in peak echo Doppler gradient indicating worsening cardiac function, and pulmonary edema are all indications of decompensation. Bed rest and B blockers to enable coronary filling are included in the treatment. In the third trimester, the majority of patients will reach a stage where the baby can be delivered without risk. Aortic valve replacement or balloon aortic valvotomy may need to be explored in extreme circumstances, albeit the latter involves a 30% risk of fetal loss [30].

A technique called an annuloplasty is used to tighten, alter, or strengthen the ring (annulus) that surrounds a heart valve. It could be carried out as part of another treatment to fix a heart valve. By performing a valvuloplasty, the heart valve's blood flow may be improved. Additionally, it could lessen chest discomfort and other heart valve disease symptoms including breathlessness.

Prosthesis valve

Due to the lack of a perfect valve, choosing a prosthetic heart valve (PHV) in women of reproductive age is still difficult. Mechanical prostheses and bioprostheses, the two main categories of artificial heart valves, both have benefits and drawbacks. Durability, the incidence of thromboembolism, and valve hemodynamics are significant areas of variance.

The three types of tissue valves (bioprostheses) are heterografts, homografts, and autografts. Women with porcine heterografts have provided the majority of the information on pregnancy in women with bioprosthetic valves. The use of tissue valves in women of reproductive age lowers the need for anticoagulation and thromboembolism prevention during pregnancy, but it is linked to a significant risk of structural valve degeneration (SVD) in young women. Numerous publications have shown conclusive evidence of pregnancy-related, accelerated tissue valve degradation.

Maternal risk

Pre-pregnancy counseling for women with congenital heart disease (CHD) should cover topics such as risks to the mother and fetus, inherited risks, potential pregnancy outcomes, sexual activity, and newborn care. It is possible that pregnant and postpartum CHD women may develop heart failure and/or arrhythmia, which will make it difficult for them to properly care for their unborn children [21]. Although the New York Heart Association (NYHA) classification is frequently used to determine whether or not pregnancy is advised, doctors should not completely depend on it to determine the likelihood of conception for each of their specific patients. Table 2 below enlists the category of patients with CHD and conditions that require strict monitoring during pregnancy or should be advised to avoid getting pregnant. High-risk CHD affects both the mother and the fetus. It may cause cyanosis to worsen, develop cardiac failure, cause arrhythmias, and may cause thromboembolism, cardiac ischemia, or aortic dissection.

**Table 2:** Conditions with increased maternal risk with the continuation of pregnancy AS = aortic stenosis; CHD = congenital heart disease; KD = Kawasaki disease; LV = left ventricle; NYHA = New York Heart Association; PH = pulmonary hypertension.

Women with conditions requiring careful monitoring during pregnancy or should avoid pregnancy
PH (Eisenmenger syndrome)
LV outflow or inflow tract stenosis (severe AS with a mean pressure gradient of >50 mmHg)
Heart failure (NYHA III to IV, LV ejection fraction <35%)
Marfan syndrome (ascending aorta diameter at end-diastole >40 mm)
Cyanotic CHD (arterial oxygenation saturation <85%)
Mechanical valves
Fontan procedure
KD with coronary artery aneurysm and stenosis
Arrhythmias that induce hemodynamic compromise

Fetal risk

Fetal and neonatal health is significantly influenced by maternal health. While still uncommon, fetal death (1.7%) and perinatal mortality (2.3%) have risen over the baseline incidence of 1% [32]. Fetal and perinatal morbidity is more prevalent, ranging from 16% to 18%, and is mainly caused by low birth weight infants (8%), preterm (16%), and prematurity-related problems (17%). Depending on the heart lesion, the probability of prenatal and neonatal problems varies. The risk of passing on CHD to kids depends on whether the mother or father has a heart problem. Transmission to kids for CHD without a hereditary condition ranges from 3% to 5%, with greater rates in aortic stenosis; left-sided outflow tracks lesions (10%) [32]. Women with diabetes also run the risk of congenital abnormalities. The majority of prenatal cardiac screening is carried out between 20 and 22 weeks of gestation, and it is best done by a person skilled in fetal cardiac imaging. Table 3 below shows the estimated fetal risks in mothers with CHDs.

**Table 3:** Fetal risks associated with congenital heart disease in mothers CHD = congenital heart disease; IUGR = intrauterine growth restriction.

Fetal risks associated with congenital heart disease in mothers
Recurrence of CHD
Preterm
IUGR
Embryopathy
Intracranial bleeding

Endocarditis

Prosthetic valve endocarditis necessitates challenging surgical procedures and occasionally produces fatal clinical outcomes, especially in individuals with early onset. Endocarditis happens about 0.5% of the time per patient every year. As a result, there is still a substantial surgical risk of PHV endocarditis, and staphylococcal species are the most frequently responsible germs [25].

Anticoagulants of choice

For both patients and doctors, managing anticoagulation therapy in pregnant women with artificial heart valves is a challenging task. It might be challenging to strike a fine balance between providing appropriate protection from a thrombotic event and protecting the unborn child. At present, there is no "optimal" anticoagulant treatment for pregnant women because there is no evidence from any clinical controlled trial; however, LMWH is used in pregnant patients. The use of vitamin K antagonists (VKAs) to prevent valve thrombosis and embolic events is the standard treatment for those who have received a mechanical heart valve [6]. However, VKAs have the potential to cross the placental barrier and cause teratogenic side effects, such as midfacial hypoplasia, stippling of the epiphyses, and abnormalities of the central nervous system, such as hydrocephalus and optic atrophy. Miscarriage and stillbirth are also possible. Even while warfarin can be harmful to the baby at any stage of pregnancy, the first trimester is when the fetus is most susceptible to teratogenic consequences. Previous case studies have shown that up to 7% of people who use warfarin during the first trimester might develop embryopathy. There is evidence in the literature that these effects are dose-dependent, and daily dosages of less than 5 mg/day seem to dramatically lower the chances of fetal toxicity.

Warfarin anticoagulation treatment may be associated with thromboembolisms, such as cerebral infarction and prosthetic valve thrombosis, and bleeding problems [8]. Intestinal lesions, atrial enlargement, and arrhythmias such as atrial fibrillation are other uses of warfarin. A history of cerebrovascular episodes prior to surgery increases the risk of thromboembolic or bleeding problems with warfarin. Per patient-year, thromboembolisms happen at a rate of around 1%, while bleeding problems happen at a rate of roughly 0.5% [18]. The data below represent different anticoagulants used in different studies (Table 4).

**Table 4:** Table showing different anticoagulants used in studies LMWH = low molecular weight heparin; UFH = unfractionated heparin; VKA = vitamin K antagonist.

Sr. No.	Author	Year of study	Name of study	Anticoagulant used
1.	Luca S De Santo et al. [4]	2011	Mechanical aortic valve replacement in young women planning on pregnancy	LMWH
2.	Snehalata Basude et al. [6]	2014	Pregnancy outcome and follow-up cardiac outcome in women with aortic valve replacement	LMWH and low-dose Aspirin
3.	Walkiria Samuel Avila et al. [7]	2003	Pregnancy in patients with heart disease: experience with 1,000 cases	Heparin
4.	Adil AM Al-Lawati et al. [8]	2002	Pregnancy and mechanical heart valves replacement; dilemma of anticoagulation	Warfarin vs. heparin
5.	Uri Elkayam et al. [9]	2005	Valvular heart disease and pregnancy	LMWH
6.	Adeline Fuchs et al. [10]	2013	Valve-in-valve and valve-in-ring transcatheter mitral valve implantation in Young women contemplating pregnancy	Aspirin
7.	Martin Sillesen et al. [11]	2010	Pregnancy with prosthetic heart valves — 30 years’ nationwide experience in Denmark	Warfarin, LMWH
8.	B Mazibuko et al. [13]	2012	An audit of pregnant women with prosthetic heart valves at a tertiary hospital in South Africa: a five-year experience	Warfarin
9.	Karen Sliwa et al. [14]	2014	Management of valvular disease in pregnancy: a global perspective	LMWH
10.	Vanita Suri et al. [15]	2011	Mechanical valve prosthesis and anticoagulation regimens in pregnancy: a tertiary center experience	UFH and LMWH
11.	Zachary L Steinberg et al. [16]	2017	Maternal and fetal outcomes of anticoagulation in pregnant women with mechanical heart valves	VKA and LMWH

Discussion

Avila et al. [7] concluded that due to the risky gestational development and lack of efficient medical resources, pregnancy is not advised in people with Eisenmenger syndrome, severe cardiomyopathies, and pulmonary vascular disease. Obtaining a positive outcome for these individuals requires both thorough clinical follow-up throughout pregnancy and proper appraisal of the mother's prognosis prior to conception.

Sillesen et al. [11] concluded that the majority of women with artificial heart valves, particularly aortic prostheses for congenital lesions, tolerate pregnancy well overall, according to extensive data collected over 30 years with various and individualized anticoagulation strategies, though the risks of cardiac mortality, anticoagulation-related mortality, and thromboembolic events are higher than in healthy controls. Increased miscarriages are the main fetal problems, along with growth restrictions that may be caused by the use of VKAs. However, warfarin embryopathy was only observed in a small number of high-risk individuals on long-term high-dose warfarin. A percentage of women with mitral valve prostheses will still need anticoagulation, despite the fact that aortic bioprostheses will definitely prevent issues linked to anticoagulation in some patients at the cost of an increased requirement for re-operations. Our statistics offer additional proof of the relevance and importance of the dangers connected to mostly warfarin-based therapy regimens, which are still elective for some patients. The statistics also provide a point of reference for freshly released series that is based on LMWH regimens.

Nanna and Stergiopoulos et al. [12] have found that since medical and surgical advancements have made it possible for many individuals with valvular heart disease to live to reproductive age, valvular heart disease in pregnancy is becoming an increasingly frequent cause of negative outcomes for both mother and baby. Rheumatic heart illness is still extremely widespread worldwide and a significant cause of VHD in immigrant populations even if it has become more uncommon in affluent nations. For pregnant women with VHD, we suggest an algorithmic approach to preconception counseling and anticoagulant therapy with WHO classification. For these challenging patients, we also suggest an early referral to a cardiologist with expertise in the treatment of cardiac disease during pregnancy.

Mazibuko et al. [13] mentioned that their study demonstrates that there is a high risk of embryopathy associated with using warfarin when pregnant. With dosages larger than 5 mg, this risk could be increased, but no firm conclusions can be made. Additionally, considerable fetal losses are linked to the use of warfarin during the second trimester of pregnancy, most likely as a result of inadequate dose monitoring and management. Maternal problems may result from switching from warfarin to heparin at the time of delivery. The American College of Cardiology/American Heart Association guidelines provide recommendations for managing anticoagulation in pregnant women with mechanical heart valve prostheses (MHVP). These recommendations are based on the views of professionals. Before definitive recommendations on an appropriate prophylactic anticoagulation regimen for the prevention of thrombosis of MHVP can be made, large randomized studies using dose-adjusted LMWH are required.

Suri et al. [15] presented the experience with various anticoagulant regimens used in a tertiary center from a developing country. Overall, 69.2% of deliveries were vaginal and 72.1% resulted in live births. Two mothers passed away with acute valvular thrombosis. In the group that took heparin in the first and late third trimesters together with acenocoumarol in the second trimester, the incidence of hemorrhagic complications was considerably greater. However, among the women on warfarin, there was a rise in the number of spontaneous miscarriages and cases of valvular thrombosis that resulted in maternal mortality.

You et al. [2] demonstrated that with the use of cardiopulmonary bypass (CPB), we were able to successfully conduct cardiac surgery on four pregnant women who had heart problems. The other two women carried on with their pregnancies following heart surgery, while two of them underwent simultaneous cardiac surgery and cesarean sections. Both mothers and fetuses of the four cases lived to adulthood. These four instances demonstrate that surgical procedures for heart disease during pregnancy with evident symptoms should be carried out effectively and proactively. Of course, the possibility of postoperative problems should be taken into consideration. An excellent strategy is the collaborative management of interdisciplinary teams.

Basude et al. [6] concluded that when compared to bioprosthetic aortic valve regurgitation (AVR) and the Ross surgery, mechanical valves were linked to somewhat high rates of maternal and fetal morbidity and mortality; however, following a brief time of follow-up, we observed no difference in valve degradation between different types of AVRs. Anticoagulation is a significant risk in pregnancy even though current-generation valves have better hemodynamics. Women of childbearing age should be informed of this before having their valves replaced.

Al-Lawati et al. [8] have shown that, since each anticoagulant regimen carries some risk, none of them can, as of yet, be deemed completely risk-free for usage during pregnancy. The decision would be made to use warfarin throughout pregnancy, including the first trimester, if the benefits and risks of each of the available anticoagulant agents were to be balanced. This is because there are nearly equal risks to the fetus with both regimens, but there are greater risks to the mother's life if heparin is used.

Elkayam and Bitar et al. [9] concluded that the decision about anticoagulation in PHV-positive pregnant women must be decided after careful consideration of the patient's wishes and those of her family. It is important to stress the potential dangers and advantages of the therapy choices that are available as well as the fact that the data currently available are insufficient to accurately predict effectiveness and safety. However, clinical experience clearly shows that the risk of anticoagulation is significantly correlated with insufficient dose and monitoring and may be significantly reduced by a strong commitment to a tight treatment regimen and regular follow-up by the patient and her doctor.

Fuchs et al. [10] reported that their limited, preliminary data imply that transseptal transcatheter mitral valve implantation (TMVI) for failed bioprostheses or rings is possible and not related to early mortality, stroke, or any other serious consequence in young women considering pregnancy. In particular, after a valve-in-valve (ViV) TMVI, it may permit uncomplicated pregnancies and postpone the final mechanical mitral valve replacement. However, following valve-in-ring (ViR) TMVI, the findings were less encouraging, and additional study is required to find the most suitable ViR candidates.

De Santo et al. [4] stated that preoperative counseling should be in-depth for young women with valvular heart disease who expect to become pregnant in the future because valve replacement surgery is still challenging for this population of patients. It is vital to have access to a broad variety of cutting-edge surgical instruments, in-depth knowledge of anticoagulant treatment protocols, and close long-term follow-up abilities to provide pregnancy management advice and ensure favorable late maternal outcomes. In this pilot observational study, the criteria helping young women scheduled for valve replacement to gain individualized information guiding the selection of prosthetic device included the right dose of warfarin from a preoperative anticoagulation trial. It is evident that this should not be the only factor taken into consideration given that women who need 5 mg per day may choose to deliver kids with mechanical valves as well as undertake heparin-based procedures in place of oral anticoagulation medication. Given the recent improvement of percutaneous techniques for valve implantation, a woman may decide to get a bioprosthesis instead and have a ViV implanted if pregnancy-related bioprosthetic degradation happens.

Sliwa et al. [14] mention that it is uncommon for pregnant patients with valvular disease to visit a particular doctor. Before becoming pregnant, it is crucial to inform the patient of the hazards associated with particular valvular disorders or types of prosthetic valves, as well as the requirement for anticoagulation. When treating pregnant patients, it is important to remember that every procedure has an effect on both the mother and the fetus. As a result, every therapeutic option needs to be maximized for both. Guidelines for the best management in a particular circumstance are based on consensus and/or opinion of subject-matter experts as well as evidence from limited prospective trials, retrospective studies, and registries since prospective or randomized studies lack the necessary data. Up until efficient prevention programs have totally eradicated rheumatic heart disease (RHD) in low- and middle-income countries (LMICs), their main issue will be access to heart valve surgery, followed by replacement valves that are suited for the young rheumatic patients of LMICs. Percutaneous valve lesion mitigation techniques, such as the MitraClip™ (Abbott, Chicago, Illinois) for mitral regurgitation or the implantation of affordable synthetic stented valves for other lesions using a nonocclusive, self-homing approach, may also offer hope for the vast majority of young women in LMICs who do not currently have access to surgery.

Steinberg et al. [16] said that VKAs are the most secure anticoagulation method for expectant moms with mechanical mitral and/or aortic valves. The negative effects of VKAs on fetal development appear to be limited to early gestation, with low rates of fetal loss and congenital malformations at warfarin levels of 5 mg daily. When compared to a VKA regimen, the usage of anti-factor Xa-adjusted LMWH is linked to higher unfavorable maternal outcomes but lower poor fetal outcomes. This is true whether the drug is used throughout pregnancy or simply during the first trimester. However, there was no distinction between people on an LMWH regimen and those on warfarin at dosages of 5 mg daily in terms of negative fetal outcomes. In comparison to a VKA regimen, the use of a UFH + VKA regimen still carries a high risk of adverse maternal outcomes without considerably lowering the risk of adverse fetal outcomes. Prospective randomized studies and big patient registry datasets are needed to validate these results.

## Conclusions

The management of pregnancy with cardiac disease operated or not operated is a huge challenge in itself. It requires vigorous screening, monitoring, and risk assessment of all the concerned factors. The perfect anticoagulants are the most demanding of all. Hence, on the basis of this review, the algorithm to manage such patients should be a thorough antenatal checkup, followed by serial echocardiograms, proper maintenance of international normalized ratio levels, use of anticoagulants, and decision of mode of delivery with a multidiagnostic approach.
